# Efficacy of Influenza Vaccination and Tamiflu® Treatment – Comparative Studies with Eurasian Swine Influenza Viruses in Pigs

**DOI:** 10.1371/journal.pone.0061597

**Published:** 2013-04-22

**Authors:** Ralf Duerrwald, Michael Schlegel, Katja Bauer, Théophile Vissiennon, Peter Wutzler, Michaela Schmidtke

**Affiliations:** 1 IDT Biologika GmbH, Dessau-Rosslau, Germany; 2 Jena University Hospital, Department of Virology and Antiviral Therapy, Jena, Germany; 3 Tierpathologie Leipzig, Leipzig, Germany; St. Jude Children's Research Hospital, United States of America

## Abstract

Recent epidemiological developments demonstrated that gene segments of swine influenza A viruses can account for antigenic changes as well as reduced drug susceptibility of pandemic influenza A viruses. This raises questions about the efficacy of preventive measures against swine influenza A viruses. Here, the protective effect of vaccination was compared with that of prophylactic Tamiflu® treatment against two Eurasian swine influenza A viruses. 11-week-old pigs were infected by aerosol nebulisation with high doses of influenza virus A/swine/Potsdam/15/1981 (H1N1/1981, heterologous challenge to H1N1 vaccine strain) and A/swine/Bakum/1832/2000 (H1N2/2000, homologous challenge to H1N2 vaccine strain) in two independent trials. In each trial (i) 10 pigs were vaccinated twice with a trivalent vaccine (RESPIPORC® FLU3; 28 and 7 days before infection), (ii) another 10 pigs received 150 mg/day of Tamiflu® for 5 days starting 12 h before infection, and (iii) 12 virus-infected pigs were left unvaccinated and untreated and served as controls. Both viruses replicated efficiently in porcine respiratory organs causing influenza with fever, dyspnoea, and pneumonia. Tamiflu® treatment as well as vaccination prevented clinical signs and significantly reduced virus shedding. Whereas after homologous challenge with H1N2/2000 no infectious virus in lung and hardly any lung inflammation were detected, the virus titre was not and the lung pathology was only partially reduced in H1N1/1981, heterologous challenged pigs. Tamiflu® application did not affect these study parameters.

In conclusion, all tested preventive measures provided protection against disease. Vaccination additionally prevented virus replication and histopathological changes in the lung of homologous challenged pigs.

## Introduction

Vaccines and antiviral drugs are essential means for control of influenza [1]. The fast spread and frequent mutation rate of influenza viruses contribute to high incidence and variability of these viruses in seasonal, epidemic, and pandemic influenza [2], [3]. The area-wide and permanent circulation of swine influenza A viruses together with the possibility of interspecies transmission and replication of avian and human influenza A viruses enables reassortment of new viruses in pigs [4]–[9]. As shown by the emergence of pandemic influenza A H1N1(2009) virus (pH1N1/2009) such reassorted viruses can represent a worldwide threat [10]–[12]. The antigenic properties as well as drug susceptibility of pH1N1/2009 are determined by gene segments of swine influenza A viruses. In particular, pH1N1/2009 became resistant to M2 channel inhibitors [13], [14] by accepting the matrix protein-coding gene of European swine influenza A viruses which confers the drug resistance [15], [16]. Since H3N2 viruses circulating in humans are also resistant to this drug class [17], [18] a situation of nearly 100% prevalence of ion channel inhibitor resistance was caused worldwide and neuraminidase inhibitors (NAI) like Tamiflu® and Relenza® are the only drugs considered for additional prophylactic use at the moment.

The current knowledge about the efficacy of existing NAI against Eurasian swine influenza A viruses is based only on *in vitro* data [19], [20]. To extend this knowledge, in the present study the efficacy of vaccination as well as the application of Tamiflu® against two Eurasian swine influenza A viruses was compared under experimental conditions in their natural host. The protective effect of vaccination was comparatively studied in a vaccine-heterologous as well a vaccine-homologous challenge.

## Results

### Comparison of efficacy of vaccination and Tamiflu® treatment against H1N1/1981 (vaccine-heterologous challenge)

H1N1/1981 had been isolated within the first period after introduction of avian-like viruses into the European pig population [21], [22]. Because the vaccine strain H1N1/2003 was isolated after 22 years of evolution of these viruses in pigs and vaccinated pigs do not cross-react in HI with H1N1/1981, challenge with H1N1/1981 allows studying the efficacy of vaccination against heterologous challenge with a not cross-reactive strain of the same influenza A virus subtype in comparison to the prophylactic effect of Tamiflu®.

**Figure 1:**
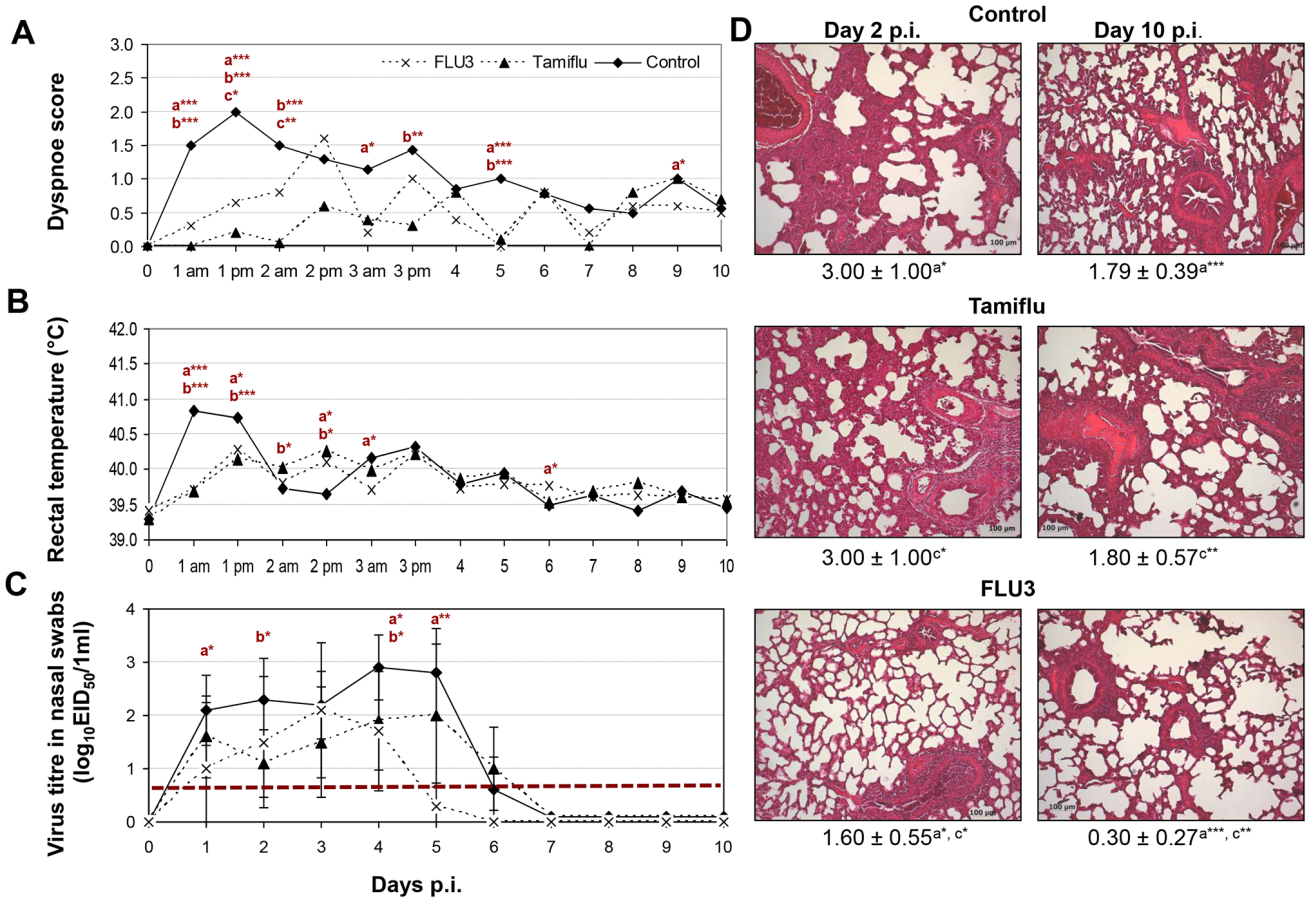
Protective effect of Tamiflu® in 11-week-old, A/swine/Potsdam/15/1981 (H1N1/1981) virus challenged pigs (n = 10) in comparison to RESPIPORC® FLU3-vaccinated (n = 10) and untreated animals (n = 12). Dyspnoea (A), rectal temperatures (B), virus titres in nasal swabs, n = 10 animals/group/day until day 2 p.i. and n = 5 from day 3 p.i. on, exception: n = 12 untreated animals at day 0 to 2 p.i. and n = 7 untreated animals/day at 3 to 7 p.i. (C), and mean of histopathological scores with standard deviations and representative photographs of formalin fixed, HE stained lungs (D) are shown (am morning; pm afternoon; p statistical probability: *p<0.05, **p<0.01, ***p<0.001, a vaccinated group versus control group, b Tamiflu®-treated group versus control group, c vaccinated group versus Tamiflu®-treated group, Mann-Whitney-U-test). The detection limit of virus titre determination is shown as dotted line (C).

Just 24 hours after infection with H1N1/1981 unvaccinated untreated pigs developed influenza with dyspnoea diagnosed until day 3 p.i. (Fig. 1A). Coughing was observed rarely in individual animals only (data not shown). Furthermore, a significant rise in body temperature was observed on day 1 p.i. (Fig. 1B). Vaccination and Tamiflu® treatment significantly reduced clinical signs (Fig. 1A and 1B). Reduction of body weight was not observed (data not shown).

**Figure 2:**
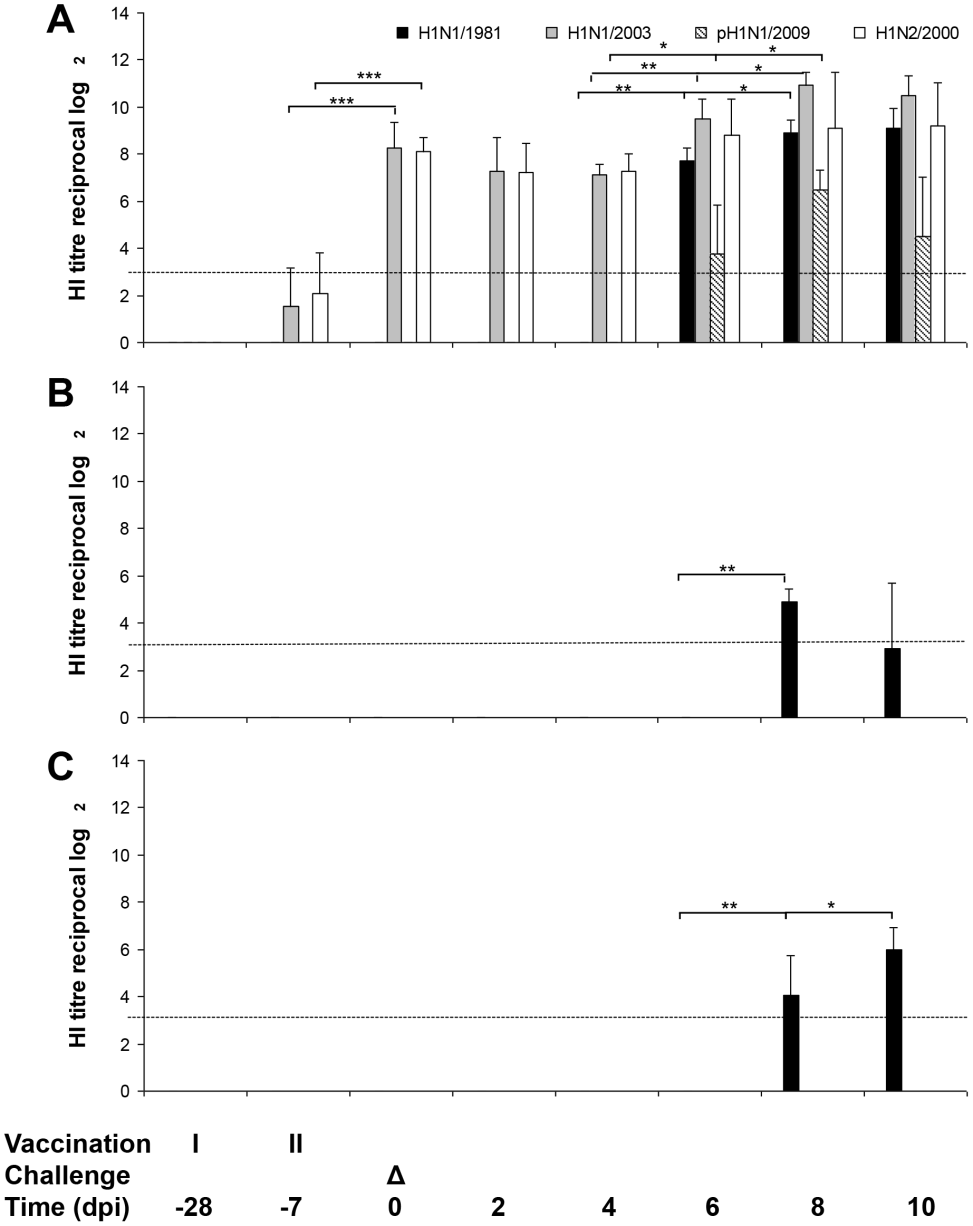
Influence of vaccination and Tamiflu® treatment on antibody kinetics in pigs challenged with A/swine/Potsdam/15/1981 (H1N1/1981) virus. Hemagglutination inhibition assays were performed with serum from pigs vaccinated with RESPIPORC® FLU3 (A), pigs treated with Tamiflu® (B), and untreated pigs (C). Geometric mean and standard deviation of antibody titres determined in serum samples of 10 vaccinated and Tamiflu®-treated or 12 control animals/ /day are shown until day 2 p.i. and 5 or 7 from day 3–10 p.i., respectively. Mann-Whitney-U-test was used to calculate p statistical probability: *p<0.05, **p<0.01, ***p<0.001. Only significant differences are shown. The detection limit of HI antibody titre determination is shown as dotted line.

Up to 6 days p.i. infected, untreated as well as Tamiflu®-treated pigs shed virus (Fig. 1C). Thereafter, virus titres decreased markedly coinciding with the appearance of first antibodies against the challenge strain (Fig. 2). All (12/12 pigs until day 2 p.i., 7/7 pigs from day 3 to 5 p.i.) untreated and unvaccinated pigs had virus titres in their nasal swabs ranging from 1.3 to 3.7 log_10_ EID_50_/ml. On day 6 p.i. 4 of 7 pigs of this group shed virus (0.9–1.3 log_10_ EID_50_/ml). On day 7 p.i. virus shedding ceased. Tamiflu®-treated pigs showed following shedding data: day 1 p.i. 9/10 pigs 1.3–2.7 log_10_ EID_50_/ml, day 2 p.i. 9/10 pigs 0.7–1.7 log_10_ EID_50_/ml, day 3 p.i. 4/5 pigs 1.3–2.7 log_10_ EID_50_/ml, day 4 p.i. 5/5 pigs 0.9–2.5 log_10_ EID_50_/ml, day 5 p.i 4/5 pigs 1.3–3.3 log_10_ EID_50_/ml, day 6 p.i. 4/5 pigs 0.7–1.7 log_10_ EID_50_/ml, day 7 p.i. 0/5 pigs. Vaccinated pigs had following shedding profile: day 1 p.i. 5/10 pigs 1.3–2.5 log_10_ EID_50_/ml, day 2 p.i. 2/10 pigs 0.7–3.3 log_10_ EID_50_/ml, day 3 p.i. 5/5 pigs 0.7–3.3 log_10_ EID_50_/ml, day 4 p.i. 3/5 pigs 1.3–2.9 log_10_ EID_50_/ml, day 5 2/5 pigs 0.7–0.9 log_10_ EID_50_/ml, day 6 p.i. 0/5 pigs, day 7 p.i. 0/5 pigs. At some time points the mean virus titre in nasal swabs of the vaccinated and the Tamiflu®-treated group was significantly lower than in the control group (Fig. 1C). However, one day after Tamiflu® treatment had been stopped, virus titres in nasal swabs increased.

On day 2 p.i, very similar, high virus load was determined in lungs of untreated, vaccinated and Tamiflu®-treated H1N1/1981-challenged pigs (Table 1). On day 10 p.i., there was no infectious virus (data not shown).

**Table 1:** Summary of the virus titres determined in left and right lung lobes and macroscopic lung lesions at ventral and dorsal view (mean ± standard deviation; n = 5; control group day 10 p.i. n = 7); on day 10 p.i. there was no virus in the lungs anymore (data not shown).

Challenge	Experimental group	Lung virus titre (log_10_EID_50_/g)		Macroscopic lung lesions (% of affected lung area) on day			
virus		on day 2 p.i.		2 p.i.		10 p.i	
		left lobe	right lobe	ventral	dorsal	ventral	dorsal
H1N1/1981	control	3.90±0.51	4.18±0.36	1.00±1.00	2.00±1.22	2.57±1.27	3.57±1.62
	Tamiflu®-treated	4.10±0.35	4.26±0.17	0.60±0.89	2.80±1.48	0.90±1.02^b^ *	1.60±1.29^b^ *
	FLU3-vaccinated	4.46±0.55	4.10±0.42	2.40±1.82	5.60±2.97^a^ *	0.60±0.82^a^ *	0.40±0.42^a^ **
H1N2/2000	control	3.54±0.62	3.30±0.20	1.90±1.52	3.00±2.32	2.71±3.30	2.86±1.95
	Tamiflu®-treated	3.70±0.62^b^ **	3.18±0.27^b^ **	0.55±0.84	0.85±1.22	0.40±0.42^b^ *	0.50±0.50^b^ *
	FLU3-vaccinated	≤0.5§ a **	≤0.5^a^ **	≤0.5^a^ *	0.05±0.11^a^ *	≤0.5^a^ **	≤0.5^a^ **

Statistics, Mann-Whitney-U test, significant differences are shown:

a , vaccinated versus control group;

b , Tamiflu®-treated versus control group; there were no significant differences concerning vaccinated versus Tamiflu®-treated group;

* p<0.05,

** p<0.01;

§ 0.5 detection limit.

Macroscopic lung lesions of control pigs mainly affected the margins of the cardiac lobes, followed by lesions on the margins of the apical lobes. The diaphragmatic lobe was only rarely affected near to the cardiac lobe. In general the extent of lung consolidation did not exceed 5–10% of the lung surface. On day 2 p.i., a protective effect was neither observed in Tamiflu®-treated nor in FLU3-vaccinated animals. Two vaccinated pigs had larger lung lesions than any other pig. On day 10 p.i., lesions were significantly lower in vaccinated pigs and Tamiflu®-treated pigs (Table 1). A histopathological score of about 3 and 2 was detected in untreated, infected as well as Tamiflu®-treated pigs on day 2 and 10 p.i. (Fig. 1D). Despite similar virus replication in the lung, a significantly lower histopathological score was observed in vaccinated pigs at both time points (Fig. 1D).

After first vaccination with FLU3 marginal antibody titres to the vaccine strains H1N1/2003 and H1N2/2000 were detected (Fig. 2A). These antibodies had risen to highly significant titres after second vaccine administration and did not differ significantly between H1N1/2003 and H1N2/2000. The challenge induced a strong H1N1/1981-specific antibody response on 6 days p.i. in vaccinated pigs whereas it was observed in pigs of the Tamiflu®-treated as well as the untreated group two days later (Fig. 2A, B, C). Vaccinated pigs developed significantly higher H1N1/1981-specific antibody titres in comparison to the Tamiflu®-treated and the untreated group on day 6, 8, and 10 p.i. (vaccinated versus Tamiflu® group: p = 0.008; vaccinated versus untreated group: p = 0.003). Starting on day 6 p.i., antibodies generated in vaccinated, H1N1/1981-infected pigs did cross-react with pH1N1/2009 (Fig. 2A). The vaccine-induced antibodies against H1N1/2003 were boosted after H1N1/1981 infection and rose significantly from day 4 p.i. to day 8 p.i. (Fig. 2A).

**Figure 3:**
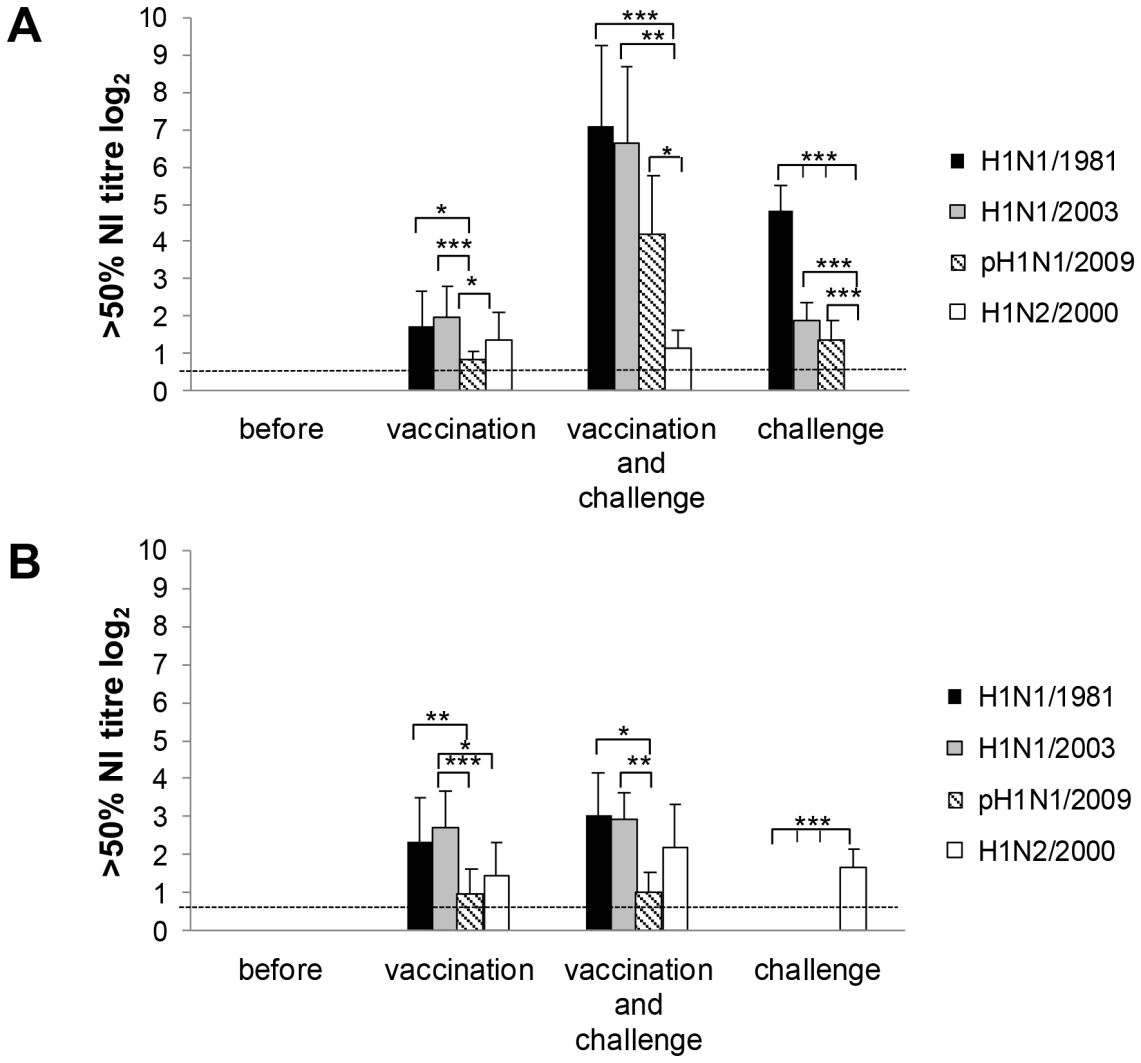
Neuraminidase inhibition by pig sera. Sera were taken shortly before vaccination, 28 days before infection (“before”), 7 days after second vaccination (“vaccination”), 14 days after second vaccination and 7 days after infection with challenge virus (“vaccination and challenge”), and 7 days after infection with H1N1/1981 in the unvaccinated, untreated control group (“challenge”). Geometric mean of antibodies which cause 50% neuraminidase inhibition with standard deviations are shown (10 pigs before and at vaccination; 5 after vaccination and challenge, and 7 after challenge) for challenge with H1N1/1981 (A) and H1N2/2000 (B). Mann-Whitney-U-test was used to calculate p statistical probability: *p<0.05, **p<0.01, ***p<0.001. Statistics are shown for comparison within the groups (only statistical differences are shown). Data of statistical analysis for comparison between the groups (“before” versus “vaccination” and so on) are given in the text under results. The detection limit of NI antibody titre determination is shown as dotted line.

The vaccine induced highly significant NI antibody titres to all tested viruses (“before” versus “vaccination”: p<0.001; for antibody titres compare with Fig. 3A; statistics for comparison between the groups are not shown in Fig. 3A). In contrast to the similar high antibody titres against H1N1/1981 and H1N1/2003, the activity against pH1N1/2009 was significantly lower. After H1N1/1981 challenge NI antibody titres to H1N1/1981, H1N1/2003, and pH1N1/2009 but not to H1N2/2000 rose significantly in vaccinated pigs (“vaccination” versus “vaccination and challenge”: p = 0.001). H1N1/1981-infected control pigs produced significantly higher NI antibody titres against the challenge strain than against H1N1/2003 and pH1N1/2009. The difference in NI antibody levels between H1N1/2003 and pH1N1/2009 after infection was not significant.

### Comparison of efficacy of influenza vaccination and Tamiflu® treatment against H1N2/2000 (vaccine-homologous challenge)

The FLU3 vaccine contains a high passage of strain H1N2/2000. Therefore, challenge with the same H1N2 enables studying the effect of vaccination against homologous virus infection.

**Figure 4:**
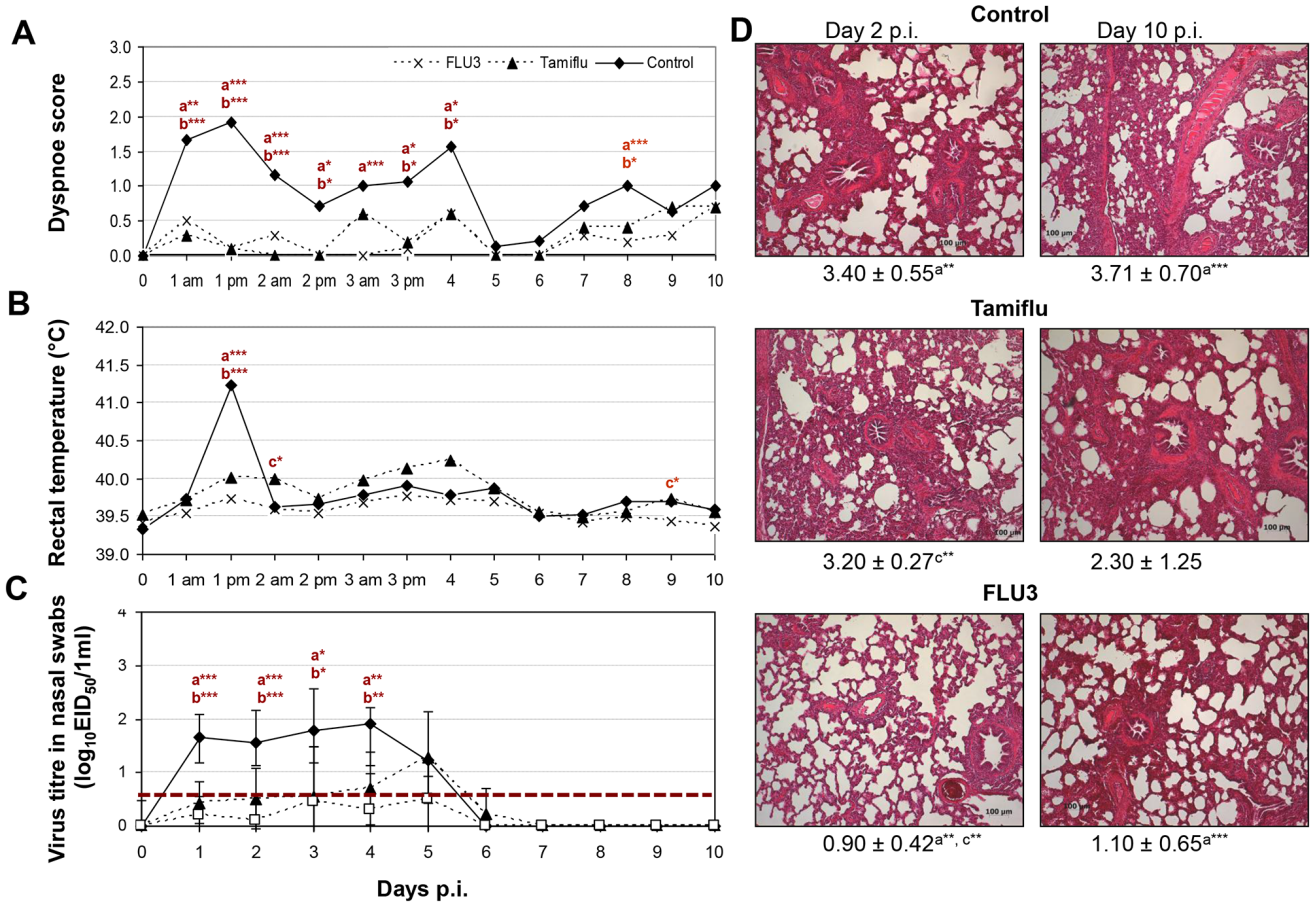
Antiviral activity of Tamiflu® in 11-week-old, A/swine/Bakum/1832/00 (H1N2/2000) virus infected pigs (n = 10) in comparison to RESPIPORC® FLU3-vaccinated (n = 10) and untreated animals (n = 12). Dyspnoea (A), rectal temperatures (B), virus titres in nasal swabs, n = 10 animals/group/day until day 2 p.i. and n = 5 from day 3 p.i. on, exception: n = 12 untreated animals at day 0 to 2 p.i. and n = 7 untreated animals/day at 3 to 7 p.i. (C), and mean of histopathological scores with standard deviations and representative photographs of formalin fixed, HE stained lungs (D) are shown (am morning; pm afternoon; p statistical probability: *p<0.05, **p<0.01, ***p<0.001, a vaccinated group versus control group, b Tamiflu®-treated group versus control group, c vaccinated group versus Tamiflu®-treated group, Mann-Whitney-U-test). The detection limit of virus titre determination is shown as dotted line (C).

Influenza induced by H1N2/2000 in untreated, infected control animals was characterized by two dyspnoea peaks on day 1 and 4 p.i (Fig. 4A) and temperature >41°C on day 1 p.i. (Fig. 4B). All pigs recovered from clinical signs within 5 days. Clinical signs were neither observed in vaccinated nor in Tamiflu®-treated pigs. None of the H1N2/2000-infected animals lost body weight (results not shown).

Vaccination as well as Tamiflu®-treatment caused a significant virus titre reduction in nasal swabs (Fig. 4C). All unvaccinated, untreated pigs shed virus from day 1 to 5 p.i. Virus titres ranged from 1.3 to 2.7 log_10_ EID_50_/ml. Mean virus titres of Tamiflu®-treated pigs were significantly reduced from 1 to 4 days p.i. (day 1 p.i. 6 of 10 pigs - 6/10 pigs - shed virus at titres ranging from 0.7–0.9 log_10_ EID_50_/ml, day 2 p.i. 5/10 pigs 0.7–1.3 log_10_ EID_50_/ml, day 3 p.i. 2/5 pigs 0.7–2.1 log_10_ EID_50_/ml, day 4 p.i. 3/5 pigs 0.9–2.3 log_10_ EID_50_/ml). After stopping Tamiflu®-treatment H1N2/2000 shedding increased in four of five pigs on day 5 (1.3–2.3 log_10_ EID_50_/ml) but vanished already 6 days p.i. due to the appearing antibodies (Fig. 4C, 5B). In contrast, five of 10 pigs of the vaccinated group did not shed the virus at all. Low virus titres of 0.7 log_10_ EID_50_/ml were detected in the nasal swabs of three vaccinated pigs 24 h p.i., from a fourth pig from day 2 to 5 p.i. (1.1, 2.3, 1.3, and 1.3 log_10_ EID_50_/ml), and in the fifth only on day 5 p.i. (1.1 log_10_ EID_50_/ml).

**Figure 5:**
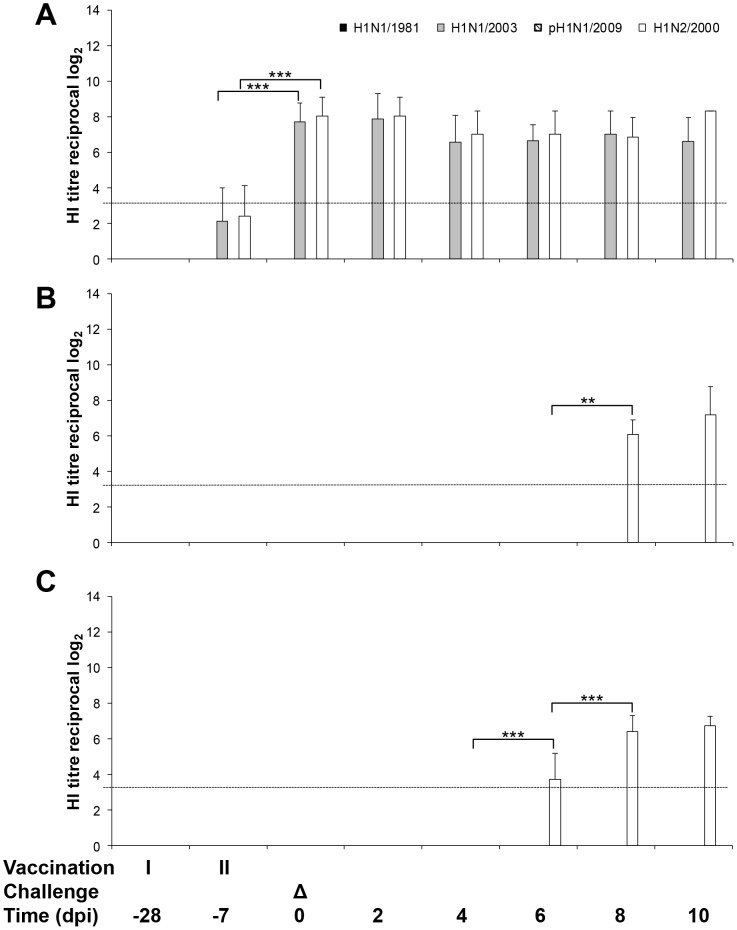
Influence of vaccination and Tamiflu® treatment on antibody kinetics in pigs challenged with A/swine/Bakum/1832/2000 (H1N2/2000) virus. HI assay was carried out with serum from pigs vaccinated with RESPIPORC® FLU3 (A), pigs treated with Tamiflu® (B), and untreated pigs (C). Geometric mean and standard deviation of antibody titres determined in serum samples of 10 vaccinated and Tamiflu®-treated or 12 control animals/day are shown until day 2 p.i. and 5 or 7 from day 3–10 p.i., respectively. Mann-Whitney-U-test was used to calculate p statistical probability: *p<0.05, **p<0.01, ***p<0.001. The detection limit of HI antibody titre determination is shown as dotted line.

While vaccination completely prevented H1N2/2000 replication in lungs of vaccinated pigs, the viral titres between the Tamiflu®-treated and untreated animals did not differ (Table 1).

The mean extent of lung consolidation of control animals challenged with H1N2/2000 ranged from 1.9 to 3.0% (Table 1). Vaccinated pigs had few or no lesions at all on day 2 and 10 p.i. A significantly reduced lung histopathology was also observed in vaccinated pigs in comparison to untreated, unvaccinated pigs on days 2 and 10 p.i. (Fig. 4D). A high mean histopathological score of about 3 was characteristic for lung tissue samples of untreated as well as Tamiflu®-treated pigs on day 2 p.i. (Fig. 4D). Whereas in control animals lung histology worsened till day 10 p.i., an improvement was observed in Tamiflu®-treated pigs (3.2 versus 2.3; not significant, Fig. 4D).

Highly significant levels of H1N1/2003- and H1N2/2000-specific HI antibodies were induced by FLU3 vaccination and not further boosted by challenge with H1N2/2000 (Fig. 5A). Antibodies to H1N2/2000 appeared in untreated (Fig. 5B) and Tamiflu®-treated (Fig. 5C) animals between day 6 and 8 p.i. No cross-reacting antibodies were detected to H1N1/1981 and pH1N1/2009. Furthermore, challenge did not affect H3N2/2003 antibody production (results not shown).

Highly significant NI antibodies were prevalent against all viruses investigated in the vaccinated group (Fig. 3B; “before” versus “vaccination”: p<0.001; statistics for comparison between the groups are not shown). The challenge with H1N2/2000 did not significantly booster these antibodies (Fig. 3B; “vaccination” versus “vaccination and challenge”: p>0.05). Significant amounts of NI antibodies were induced against N2 in the unvaccinated, untreated control group (Fig. 3B; “before” versus “challenge”: p<0.001). They did not act against N1.

## Discussion

High-dose aerosol infection of pigs with H1N1/1981 and H1N2/2000 caused sudden onset of high fever and dyspnoea like influenza in humans [2], [3]. Compared with pig infection trials reported so far the observed clinical symptoms were stronger [23]–[28]. These similarities between influenza in aerosol-infection pig models and influenza in humans and pigs in addition to the similar disease course induced by H1N1/1981 and H1N2/2000 provided a good basis for comparatively evaluating the efficacy of vaccination against heterologous and homologous challenge as well as NAI treatment in the present study. The results reveal different degrees of protection.

Like in humans [29], [30], (i) vaccination as well as Tamiflu®-treatment significantly reduced clinical symptoms and virus shedding whereas (ii) vaccination was less effective when the challenge occurred with heterologous H1N1/1981 than with homologous H1N2/2000. The faster and stronger antibody response against the heterologous challenge strain H1N1/1981 may explain the efficacy of vaccination in the absence of virus-specific HI antibodies against the challenge virus. It suggests a certain degree of reactivity between older and more recent H1N1 strains. The latter could also account for detection of cross-protecting HI antibodies against pH1N1/2009 that concurs strongly with recently published studies [31]–[34]. Moreover, a higher antibody response against the vaccine strain H1N1/2003 was detected after H1N1/1981 challenge reflecting the “antigenic sin” [35], [36]. Additionally, NI can contribute to the protective effect seen after vaccination and heterologous challenge. N1 cross-reacting NI antibodies were detected indicating that neuraminidases of H1N1 strains are still antigenetically related to each other. Moreover, major histocompatibility complex restricted epitopes conserved in nucleoprotein and matrix protein could be involved in protection as discussed for human seasonal influenza A viruses and pH1N1/2009 virus [37]. European swine influenza A viruses share similar nucleoprotein, matrix, and polymerase genes.

Marked differences were found comparing the efficacy of the studied preventive measures regarding lung viral load, macroscopic lesions, and inflammation. The lack of virus inhibition in the lung after vaccination and heterologous challenge reflects the pathogenic role of antigenetic drift in European swine Influenza A (H1N1) viruses between 1981 and 2003. It also demonstrates a low efficacy of Tamiflu® regarding this study parameter. Macroscopic lung lesions were almost absent after vaccination and homologous challenge. They were also reduced by Tamiflu® treatment as well as in vaccinated, heterologous challenged pigs on day 10 p.i. after primary enhancement on day 2 p.i. Based on similar observations until day 5 p.i., Gauger et al. postulated that vaccination may potentiate influenza following challenge with divergent homosubtypic viruses that do not share cross-reacting hemagglutinin or serum neutralizing antibodies [32]. But, the significant reduced lung consolidation on day 10 p.i. reported here suggests that this effect is transient and reversed by antibodies specific to the challenge virus. With regard to inflammation, a significant score reduction was found after vaccination but not after drug treatment. Taken together, these results suggest that the pathogenetic processes which lead to induction of disease are blocked at different stages by vaccination and Tamiflu®. The latter prevented disease despite high viral lung load and interstitial lymphoid tissue hyperplasia.

Two further aspects should be mentioned concerning Tamiflu® treatment. First, the increased virus shedding one day after drug cessation suggest a need for prolonged treatment of pigs until the appearance of protective antibodies in serum. Detection of virus-specific protective antibodies in the blood correlating with virus clearance in the nose underlines this conclusion. Secondly, H1N2/2000 but not H1N1/1981 is additionally glycosylated at Asn163 in the HA and NAI-resistant in cell culture [19]. *G*lycosylation in position 163 of HA hampers the HA-NA balance and reduces NAI efficacy *in vitro* [38]. In contrast to *in vitro* results however, the prevention of influenza in pigs indicates that HA glycosylation at Asn163 does not necessarily affect the efficacy of Tamiflu® in the natural host.

In summary, due to the high similarity in the course of influenza A virus infection in pigs and seasonal influenza in humans, the pig infection model described here provides a valuable tool for antiviral investigations. In comparison of the tested preventive measures (vaccination and treatment with Tamiflu®), all protected against disease. Vaccination provided the most optimal protection at homologous challenge. Here, virus replication and histopathological changes in the lung were prevented.

## Materials and Methods

### Ethics Statement

All trial procedures and animal care activities were conducted in accordance with the guidelines and under approval of Good Clinical Practice (VICH GL9, CVMP/VICH/595/98), the Directive 2001/82/EC on the Community code relating to veterinary medicinal products and German Animal Protection Law. The protocol IDT A 3/2004 was approved by the Landesverwaltungsamt Sachsen-Anhalt (Reference Number: AZ 42502-3-401 IDT).

### Cells and Viruses

Madin-Darby canine kidney (MDCK) cells (Friedrich-Loeffler Institute, Riems, Germany) were grown in modified Eagle minimum essential medium (MEM-FLU3, IDT Biologika GmbH, Dessau-Roßlau, Germany) supplemented with 5% fetal bovine serum (Biochrom AG, Berlin, Germany).

Influenza viruses A/swine/Potsdam/15/1981 (avian-like H1N1; H1N1/1981) and A/swine/Bakum/1832/2000 (human-like H1N2; H1N2/2000) (Federal Institute for Risk Assessment, Berlin, Germany, [5]) had been isolated from pig herds in Germany during clinical outbreaks. Additionally, pandemic influenza virus A/Jena/VI5258/2009 (pH1N1/2009) was included in serological studies (Jena University Hospital, Germany). Virus cultivation in MDCK cells was supported by adding 4 Nα-benzoyl-L-arginine ethyl ester units trypsin (Sigma Aldrich, Taufkirchen, Germany) to 1 ml MEM-FLU3.

### Animals

64 crossbred swine (Piétrain×Large White; 48 males and 16 females; IDT Biologika GmbH, Dessau-Roßlau, Germany) born on the same farrowing occasion were used in the present study. Pigs had been proved to be free of influenza during their life span as well as free of maternally-derived antibodies against pH1N1/2009, avian H1N1 and human H1N2 influenza A viruses. They were housed in identical isolation rooms based on their challenge status and were provided with feed and water ad libitum.

### Compounds

Commercially available Tamiflu® capsules (F. Hoffmann-La Roche AG, Basel, CH, batch B113313, 75 mg oseltamivir per capsule) were used for *in vivo* antiviral studies according to summary of product characteristics.

### Vaccine

The trivalent inactivated swine influenza A virus vaccine RESPIPORC® FLU3 (FLU3, IDT Biologika GmbH, Dessau-Rosslau, Germany; Batch 0050806) was used for vaccination of pigs. It contained the highly passaged vaccine strains A/swine/Bakum/IDT1769/2003 (H3N2/2003), H1N2/2000, and A/swine/Haseluenne/IDT2617/2003 (H1N1/2003), carbomer 971 P NF (0.998 mg/ml) as adjuvant and thiomersal (0.095 mg/ml) for preservation. In the batch potency testing the guinea pig geometric means of neutralizing units were 11.07 for H1N1/2003, 14.84 for H1N2/2000, and 12.67 for H3N2/2003. FLU3 had a pH of 7.1, was sterile, free of extraneous viruses and complied with the requirements for release.

### Experimental Design

#### Group classification and experimental conditions

Two independent trials, one with H1N1/1981 (heterologous challenge with homosubtypic virus not cross-reactive to sera of vaccinated pigs) and another with H1N2/2000 (homologous challenge with the same strain as in the vaccine highly cross-reactive to sera of vaccinated pigs) were performed. The experimental design is summarized in Table 2. In each trial 32 pigs were allotted randomly into 3 groups. One group of 10 pigs was vaccinated i.m. with 2.0 ml of FLU3 21 and 7 days before challenge. Another group of 10 pigs was treated orally with Tamiflu® starting with 2 capsules the evening before challenge. Then, 2 Tamiflu® capsules were administered twice daily for 4 days. The third group included 12 unvaccinated untreated pigs as control.

**Table 2:** Overview of the experimental design of the study.

Challenge virus	Group	Number of pigs	Treatment
A/sw/Potsdam/15/1981 (avH1N1):	1	10	FLU3* vaccination on day 28 and 7 before challenge
heterologous	2	10	Treatment with 150 mg of oseltamivir ** the evening before challenge and 2 times 75 mg/day on the 4 following days
	3	12	None (untreated, unvaccinated control)
A/sw/Bakum/1832/2000 (huH1N2):	4	10	FLU3* vaccination on day 28 and 7 before challenge
homologous	5	10	Treatment with 150 mg of oseltamivir ** the evening before challenge and 2 times 75 mg/day on the 4 following days
	6	12	None (untreated, unvaccinated control)

* RESPIPORC® FLU3 vaccine contains following inactivated viruses: A/sw/Haseluenne/IDT2617/2003 (avH1N1), A/sw/Bakum/1832/2000 (huH1N2), and A/sw/Bakum/IDT1769/2003 (huH3N2);

** Tamiflu®.

At an age of 11 weeks, pigs of all 3 groups were simultaneously challenged by one-hour-aerosol exposure. Aerosols of H1N1/1981 and H1N2/2000 were dispersed through a flow aerosol generator which produces droplets of 0.5 to 20 µm under atmospheric pressure. H1N1/1981 was nebulised at a dose of 10^7.85^ TCID_50_/m^3^ and H1N2/2000 at a dose of 10^7.33^ TCID_50_/m^3^.

Experimental infections were done in BSL-2 infection units with High Efficiency Particulate Airfilter H13 filters.

#### Study Parameters and Sampling

After infection, rectal temperatures and signs of respiratory disease, dyspnoea, and cough were recorded twice daily 1–3 days p.i. and daily from 4–10 days p.i. Dyspnoea was assessed as follows: 1, increased respiratory frequency and moderate flank movement; 2, marked breathing difficulty and severe flank movement; 3, laboured breathing affecting the entire body, pronounced flank movement and substantial movements of the snout, 4, extreme breathing difficulty reflecting substantial lack of oxygen. Body weights were recorded daily. Nasal swab samples were collected daily in 2.0 ml stabilisation medium containing 60 ml Dextran-Sucrose-Glutamate solution (DSG 72: 126 g dextran 40, 1,5 kg sucrose, 3,6 g potassium-L-glutamate-monohydrate, 5 g potassium-dihydrogen-phosphate, 12,5 g potassium-monohydrogen-phosphate, made up to 10 l with water ad injectionem, IDT Biologika GmbH, Dessau-Rosslau, Germany, internal use), 0.2 ml gentamycin (Fagron GmbH, Barsbüttel, Germany), 2 ml amphotericin B (Sigma-Aldrich, Taufkirchen, Germany), made up to 200 ml with cell culture medium (IDT Biologika GmbH, Dessau-Roßlau, Germany, internal use).

In each trial 5 animals of each group were stunned by electrical stunning tongs 2 days p.i. and bled to death. On 10 days p.i. the remaining animals were slaughtered in the same way.

Lung tissue samples were taken from each lobe for virus detection. Samples of the right and left halves of the lungs were pooled, ground with sterile sea sand, and diluted 1∶10 in dilution medium (1.0 ml Amphotericin B and 0.1 ml Gentamycin, made up to 100 ml with phosphate buffered saline solution). Additionally, lung tissue was collected and fixed in 10% neutral buffered formalin for histopathological evaluation.

Blood samples for immunological analysis were taken immediately before the first and second vaccinations, 7 days after the second vaccination (before challenge), and 2, 4, 6, 8, and 10 days p.i.

### Hemagglutination Inhibition (HI) Assay

Sera were pre-treated with neuraminidase (Sigma, EC3.2.1.18 Type IV from *Clostridium perfringens*, 14–18 h at 37°C). After adding sodium citrate (1.5%) inactivation was carried out (30 minutes at 56°C), followed by adsorption to chicken erythrocytes (1 h at 4–8°C).

8 hemagglutinating units (HU) of the 3 vaccine strains, H1N1/1981, and pH1N1/2009 were used as antigens and incubated with 1∶10 prediluted sera in microtitre plates for 30 min at room temperature. Then a 0.5% chicken erythrocyte suspension was added and incubated for 30 min at room temperature.

### Determination of 50% Egg Infectious Dose (EID_50_)

Dilution series (log_10_) from both lung and nasal swab samples were injected into the allantois cavity of 11-day-old chicken embryos (0.1 ml; 5 eggs per dilution). After sealing the perforation point eggs were incubated at 37°C and checked daily for vitality using an egg candler. On day 4 p.i., the allantois fluid was collected and tested in the hemagglutination test. The Spearman and Kaerber method was used to calculate the EID_50_ from the hemagglutinating activity [39], [40]. The detection limit was 0.7 log_10_ EID_50_/ml.

### NA Inhibition (NI)

NI was analyzed using modified protocol of Sandbulte et al. [41]. Briefly, OD was measured at wavelength 550 nm. For data analysis, the absorbance of the fetuin control wells was subtracted from the OD values and the dilution of sera that resulted in a reading equal to 50% of the positive control (virus, no serum) was determined. The inverse of this dilution was the NI titre. Assay validity was supported by positive control samples (virus+fetuin) with mean absorbance of 0.7–1.3, negative control samples (fetuin only) with mean absorbance <0.08, and control serum which did not significantly inhibit NA activity.

### Lung Pathology and Histopathology

The pathology of the lungs was evaluated macroscopically, photographs were taken, and observed lesions were recorded onto a lung diagram. Percentage of affected lung surface area was assessed for each lobe at dorsal and ventral view.

Formalin-fixed lung tissue samples were embedded in paraffin. 5 µm-thick sections were stained with haematoxylin and eosin for light microscopy. Inflammation was scored on a semi quantitative scale from 0–4: 0, no inflammation; 1, discreet interstitial alveolar macrophages; 2, slight interstitial bronchial associated lymphoid tissue hyperplasia; 3, distinct interstitial alveolar macrophages; 4, distinct interstitial and massive broncholuminal alveolar macrophages.

### Statistical Analysis

Mann-Whitney-U-test was performed to evaluate statistical significances.
